# Laser Treatment for Patients With Vulvodynia and Interstitial Cystitis/Bladder Pain Syndrome: A Case Series (The UNICORN-3 Study)

**DOI:** 10.7759/cureus.41786

**Published:** 2023-07-12

**Authors:** Nobuo Okui, Machiko Aurora Okui, Yuko Kouno, Kaori Nakano

**Affiliations:** 1 Dentistry, Kanagawa Dental University, Kanagawa, JPN; 2 Urogynecology, Yokosuka Urogynecology Clinic, Kanagawa, JPN; 3 Urology, Dr Okui's Urogynecology and Urology, Yokosuka, JPN

**Keywords:** ai chatbot gpt-4, ai & robotics in healthcare, interstitial cystitis symptom index and problem index, pelvic pain and urgency/frequency symptom score, numeric rating scale, neodymium:yag laser, erbium:yag laser, vulvodynia, interstitial cystitis/bladder pain syndrome

## Abstract

Introduction

Interstitial cystitis/bladder pain syndrome (IC/BPS) is a chronic pain disorder characterized by urgency, frequency of urination, and pelvic pain. Women with IC/BPS often experience sexual dysfunction, vulvodynia, and vaginal health issues. Combined erbium and neodymium yttrium aluminum garnet (YAG) laser treatments targeting the vagina and vulva have shown promise in improving symptoms. Our study aims to investigate the effectiveness of these combined laser treatments in women with IC/BPS and vulvodynia.

Methods

Women diagnosed with vulvodynia and IC/BPS underwent combined laser treatment using vaginal erbium:YAG laser (VEL) and neodymium:YAG laser (Nd:YAG). Various parameters were evaluated, including the vulvodynia test, numeric rating scale (NRS-11) for pain, interstitial cystitis symptom index and problem index (ICSI and ICPI), pelvic pain and urgency/frequency symptom score (PUF), and mean urination volume/daily urination frequency in a three-day urination diary. Treatment was administered three times, with intervals of one month between each session, and follow-up evaluations were conducted at six and 12 months. All statistical analyses were designed and programmed by the AI chatbot GPT-4 (chatGPT-4).

Results

Fifteen female patients diagnosed with vulvodynia and IC/BPS were treated with three sessions of VEL + Nd:YAG. Significant improvements were observed in the vulvodynia test, NRS-11 scores, PUF, ICSI scores, ICPI scores, mean urination volume, and daily urination frequency at six and 12 months (p<0.01). Short-term improvements in IC/BPS pain scores correlated with improvements in the vulvodynia test (p=0.007), suggesting a synergistic effect. However, no significant correlations were found at 12 months.

Conclusion

Combined laser treatments targeting the vagina and vulva showed significant therapeutic effects in women with IC/BPS and vulvodynia. The addition of Nd:YAG to the VEL treatment enhanced outcomes. Short-term improvements in IC/BPS pain scores correlated with improvements in the vulvodynia test, indicating a synergistic effect. Long-term improvements in both vulvodynia and IC/BPS symptoms may occur independently. These findings highlight the importance of comprehensive approaches for treating coexisting vulvodynia and IC/BPS.

## Introduction

Interstitial cystitis/bladder pain syndrome (IC/BPS) is a chronic pain disorder characterized by symptoms such as urgency and frequency of urination, typically confined to the pelvic organs, pelvic floor musculofascial support, or external genitalia [1,2]. Despite various therapeutic approaches being proposed, there remains no definitive treatment. IC/BPS is defined based on patient reports and presents a broad range of clinical symptoms due to various underlying causes, suggesting the possible existence of fundamentally different etiological subgroups [3].

Recent studies have reported that women with IC/BPS show more significant sexual dysfunction, deteriorated vaginal health status, and increased vulvodynia (persistent unexplained pain in the vulva of more than three months duration) compared to healthy individuals [4,5]. Vulvodynia and IC/BPS often co-occur and are considered synergistic syndromes [4,5]. Given the limited efficacy of direct treatments for the bladder in IC/BPS women, combined treatment approaches targeting the vagina and vulva have been contemplated [4,5].

Initial reports have shown encouraging results with the use of vaginal non-ablative erbium: yttrium aluminum garnet (Er:YAG) laser therapy (VEL) [6], and subsequent studies have demonstrated improvements in vaginal health status with transvaginal photobiomodulation [7]. There have also been case reports of concurrent improvements in vulvodynia and Hunner's ulcers in women with IC/BPS using combined VEL and neodymium:YAG (Nd:YAG) laser treatments for the vagina and vulva [8]. In our study, we seek to explore the effectiveness of these combined laser treatments in a cohort of women, through a case series involving 15 participants.

## Materials and methods

Study design

This retrospective case series followed the journal's policy. Ethics Committee approval was obtained from the Ethical Review Board of the Kanagawa Association of Medical and Dental Practitioners (approval number 22002). Our study, named the UNICORN-3 study, was conducted as part of the UNICORN-study [6], established with the purpose of evaluating laser treatments. All patients opted in via the study's homepage [9] and gave their informed consent with a signature after being fully informed.

Patients

Fifteen female patients who visited our hospital from 2017 to 2020 and were diagnosed with vulvodynia and IC/BPS were included. All patients had previously been diagnosed with IC/BPS at other hospitals and had undergone multiple treatments according to Japanese guidelines [10]. However, the effect of these treatments lasted only a few months. At our hospital, we confirmed the diagnosis of IC/BPS based on bladder hydrodistension according to the guidelines of the Japanese Society for Interstitial Cystitis [10]. Vulvodynia is not widely recognized in Japan, and as a result, the diagnosis was made at our hospital. Despite administering three months of local estrogen therapy (LET) [8] to all cases of vulvodynia, no observed improvement was seen in the patients.

Inclusion criteria

Among the registered patients, we included women who underwent a total of three laser treatment sessions administered once a month, and we had treatment records available for one year after the final laser treatment. For IC/BPS, inclusion criteria were as follows: no improvement for three months or more despite multiple treatments targeting the bladder, and no improvement in vulvodynia after three months of LET [8]. Additionally, patients who did not experience pain relief with oral medication or anti-inflammatory analgesics were included. Conditions commonly associated with IC/BPS and vulvodynia, such as irritable bowel syndrome [11,12], fibromyalgia [12], systemic lupus erythematosus [13,14], and chronic fatigue syndrome [12], were also included in the study.

Exclusion criteria

Pregnant, breastfeeding, or women suspected of being pregnant were excluded. Also excluded were patients with urinary tract infections (confirmed by urine culture in the past six months), those on prophylactic microbial therapy, bladder intravesical chemotherapy/immunotherapy, and pelvic radiation therapy. Patients with urinary tuberculosis, and those with hematuria who had not undergone thorough examination until that day were excluded. Women with creatinine clearance levels less than 30 ml/min as measured and those with severe motor neuron or spinal diseases were also excluded.

Genital and bladder pain evaluation

Patients were subjected to the vulvodynia test [5], a baseline screening for vulvar pain where several points in the vulvar region are stimulated with a cotton swab and rated on a scale from 0 (no pain) to 10 (maximum pain). For our study, we took pain from four equidistant points around the hymen and averaged those scores. The numeric rating scale (NRS-11) score [10] for IC/BPS had to be four or above. NRS-11 also ranges from 0 (no pain) to 10 (maximum pain). It's used as a baseline screening for bladder, urethral, and perineal pain due to bladder filling. Pain intensity was measured in two situations: when the bladder was full and at the moment when the patient had to urinate due to unbearable pain. Both measurements were taken into account, and the higher value between the two was considered for analysis.

Other evaluations

The interstitial cystitis symptom index and problem index (ICSI, ICPI) were used to investigate the frequency of urination, urgency, nocturia, and bladder pain, as well as the degree of the problem [14]. The ICSI ranges from 0 to 20, and the ICPI from 0 to 16. The pelvic pain and urgency/frequency symptom score (PUF) was used, which focuses on questions about frequency of urination, urgency, pain during intercourse, and pain in the bladder, urethra, and vulva, and ranges from 0 to 35. A three-day urination diary was kept for the number and volume of urinations per day [15].

Treatment

The combined VEL and Nd:YAG laser (VEL + Nd:YAG) treatments were conducted in an outpatient setting. Before the procedure, the patient's vagina was washed with a disinfectant and dried with a cotton swab. The RenovaLase® VEL protocol was used at a wavelength of 2,940 nm. As previously reported, to prevent tissue ablation due to deep thermal effects, the laser spot diameter was set to 7 mm, frequency to 1.6 Hz, and pulse fluence to 1.75 J/cm2 [6,7]. After VEL treatment, the Nd:YAG laser (Fotona SP Dynamis, PIANO mode, spot size 9 mm, R33 non-contact handpiece, PIANO pulse mode (5 seconds), fluence 90 J/cm^2^) was used. Patients were advised to avoid sexual intercourse for one week after each session [6,7].

Treatment schedule

After three months of LET, which didn’t produce any improvement, the first visit for laser treatment (T0), VEL + Nd:YAG was administered once a month for three months. After the final laser treatment, follow-ups were conducted at six months (T06) and 12 months (T12). Evaluations were conducted at T0, T06, and T12.

Statistical methods

Statistical data were submitted to AI chatbot GPT-4 (chatGPT-4) for the suggestion of appropriate statistical methods. The chatbot proposed the use of Pearson’s correlation and t-tests. To evaluate the treatment effect, it suggested calculating changes from T0 to T06 (Δ_T0-6_) and from T0 to T12 (Δ_T0-12_) and determining the correlation of each factor with the vulvodynia test. All suggestions made by chatGPT-4 were formulated into R language codes. The raw data was then input into the coded program. Significance was set at a level less than 0.05.

## Results

During the registration period, 15 patients were diagnosed with both vulvodynia and IC/BPS and underwent VEL + Nd:YAG laser treatment. None of the patients missed a minimum of two follow-up visits after the procedure. Out of the 15 patients included in our study, two were diagnosed with IC/BPS presenting Hunner’s lesions, and 13 were diagnosed with non-Hunner-lesion IC/BPS. Two patients recognized that vulvodynia was a more severe pain than IC/BPS while the remainder reported both conditions to be an equal cause of their pain.

Table 1 investigated the correlation between each parameter (age in years, BMI in kg/m^2^, smoking, hypertension, diabetes mellitus, alcohol consumption of more than four days per week, duration of IC treatment in years) and the vulvodynia test or the NRS-11. No correlation was found in any of the items. None of the women participating in our study had conditions such as systemic lupus erythematosus or fibromyalgia, and none had a diagnosis of irritable bowel syndrome.

**Table 1:** Characteristics of patients NRS-11: The Numeric Rating Scale; BMI: Body Mass Index

	Mean±SD, number (percentage%)	NRS-11		Vulvodynia test	
		Correlation coefficient (95% confidence interval)	p-value	Correlation coefficient (95% confidence interval)	p-value
Age (years old)	59±8.14	0.053 (-0.472-0.55)	0.851	-0.148 (-0.614-0.394)	0.598
BMI (kg/m^2^)	22.9±1.69	0.052 (-0.472-0.55)	0.851	0.0612 (-0.466-0.556)	0.829
Smoking	3 (20%)	-0.162 (-0.622-0.382)	0.565	0.302 (-0.249-0.705)	0.275
Hypertension	6 (40%)	-0.044 (-0.544-0.479)	0.876	-0.123 (-0.598-0.415)	0.662
Diabetes mellitus	0	-	-	-	-
Alcohol (> 4 day /week)	5 (33%)	0.0762 (-0.454-0.566)	0.787	0.426 (-0.11-0.77)	0.113
Duration of IC treatment (years)	2.93±1.18	0.505 (-0.00968-0.808)	0.0548	0.0936 (-0.44-0.578)	0.74

**Figure 1:**
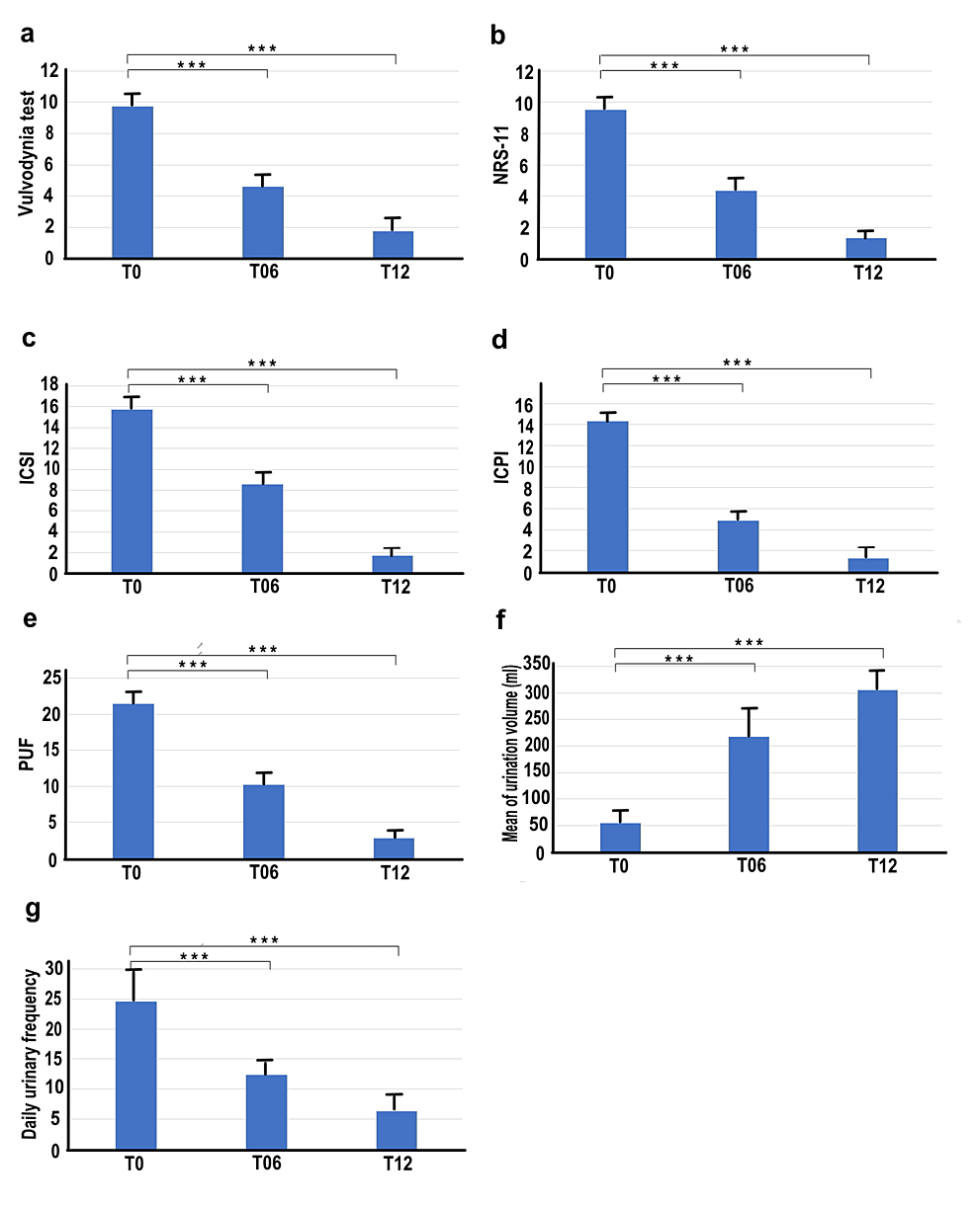
Changes in parameters over time in women with IC/BPS and vulvodynia NRS-11: The Numeric Rating Scale for Bladder Pain ICSI: The Interstitial Cystitis Symptom Index ICPI: The Interstitial Cystitis Problem Index PUF: Pelvic Pain and Urgency/Frequency Symptom Score T0: before laser treatment T06: 6 months after laser treatment T12: 12 months after laser treatment The vertical axis represents each parameter (a: Vulvodynia test, b: NRS-11, c: ICSI, d: ICPI, e: PUF, f: mean urination volume, g: daily urination frequency) and the horizontal axis represents the time points (T0, T06, T12). "Mean of urination volume" is measured in milliliters. If there was a significant difference (p<0.001) in the change from T0 to T06 or from T0 to T12, an asterisk (***) is marked on the graph.

Figure 1 showed the parameters at T0, T06, and T12. The vulvodynia test scores initially registered at 9.73±0.44 at T0, reduced to 4.6±0.48 six months post-treatment (T06), and further decreased to 1.73±0.68 at 12 months (T12). Significant differences were detected between T0 and T06, as well as T0 and T12 (p<0.001 in both instances) (Figure 1a).

In the case of bladder pain, as measured by the NRS-11 scale, the score of 9.53±0.62 at T0 diminished to 4.33±0.47 at T06 and further to 1.33±0.47 at T12. Notable differences were observed between T0 and T06, and T0 and T12 (p<0.001 in both instances) (Figure 1b).

For ICSI, scores were 15.73±1.77 at T0, reduced to 8.53±3.20 at T06, and further to 1.73±0.77 at T12. Significant differences were noted between T0 and T06, and T0 and T12 (p<0.001 in both cases) (Figure 1c).

Regarding ICPI, the scores of 14.33±1.07 at T0 decreased to 4.87±1.31 at T06, and to 1.26±0.92 at T12. Significant differences were seen between T0 and T06, as well as T0 and T12 (p<0.001 in both instances) (Figure 1d).

Additionally, for the PUF, the average scores were 21.47±1.31 at T0, decreased to 10.19±1.47 at T06, and further to 2.8±0.75 at T12. Notable differences were observed between T0 and T06, and T0 and T12 (p<0.001 in both instances) (Figure 1e).

In the case of the mean urination volume, the measurements were 54.13±19.29ml at T0, increased to 217.67±54.49ml at T06, and further to 305.53±40.93ml at T12. Significant differences were recorded between T0 and T06, and T0 and T12 (p<0.001 in both cases) (Figure 1f).

For daily urination frequency, the scores were 24.6±6.58 at T0, which decreased to 12.33±2.91 at T06, and further to 6.33±1.49 at T12. Notable differences were observed between T0 and T06, and T0 and T12 (p<0.001 in both cases) (Figure 1g).

Table 2 investigates the changes from T0 to T06 (Δ_T0-6_) and from T0 to T12 (Δ_T0-12_), evaluating whether treating the external genitalia has a direct impact on the bladder. Upon calculating the correlation between Δ_T0-6_ and Δ_T0-12_ in the Vulvodynia test and respective parameters, only the Δ_T0-6_ of NRS-11 and PUF showed significant correlation with the Δ_T0-6_ in the Vulvodynia test, while other parameters did not. Moreover, in Δ_T0-12_, the Vulvodynia test did not show any correlation with any parameters.

**Table 2:** Correlation of Vulvodynia Test and Parameter Changes NRS-11: The Numeric Rating Scale for Bladder Pain ICSI: The Interstitial Cystitis Symptom Index ICPI: The Interstitial Cystitis Problem Index PUF: The Pelvic Pain and Urgency/Frequency Symptom Score ΔT0-6: the change from T0 to T06 ΔT0-12: the change from T0 to T12 T0: before laser treatment, T06: 6 months after laser treatment T12: 12 months after laser treatment

	Vulvodynia test			
	ΔT0-6		ΔT0-12	
	Correlation coefficient (95% confidence interval)	p-value	Correlation coefficient (95% confidence interval)	p-value
NRS-11	0.663(0.228-0.877)	0.007	0.28 (-0.272-0.693)	0.313
ICSI	-0.272 (-0.689-0.279)	0.326	-0.12 (-0.596-0.418)	0.67
ICPI	-0.159 (-0.621-0.384)	0.571	0.0636 (-0.464-0.558)	0.822
PUF	0.589 (0.11-0.846)	0.020	-0.14 (-0.609-0.401)	0.618
Frequency of urination	-0.00805 (-0.518-0.506)	0.977	-0.157 (-0.619-0.386)	0.576
Capacity of bladder	0.0431 (-0.48-0.543)	0.879	0 (-0.512-0.512)	1.00

The side effects from the VEL + Nd:YAG laser treatment included sensations of heat and pain during treatment, which quickly dissipated. No cases of infection such as cystitis were observed.

Supplemental data

The thought process suggested by chatGPT-4 was as follows: Upon presenting the overall outline of the study, chatGPT-4 proposed conducting a t-test to assess the significance of the data obtained, divided into two sets: T0 to T6 and T0 to T12. Next, it provided two instructions: (1) Since the focus of this study is on pain, it recommended performing statistical analysis to confirm that the vulvodynia test, which evaluates pain in the genitals, and NRS-11, which evaluates pain in the bladder, have no correlation with the patient's baseline information. (2) Considering that laser treatment is targeted at the genitals, it suggested conducting statistical analysis to examine the correlation between various factors (NRS-11, ICSI, ICPI, PUF, frequency of urination, capacity of the bladder) and the Δ_T0-6_ and Δ_T0-12_ of the Vulvodynia test. As there was no correlation in (1), it proposed using the Pearson correlation coefficient for the statistical analysis in (2) as it would be appropriate.

## Discussion

In our study, 15 patients diagnosed with Vulvodynia and IC/BPS underwent VEL + Nd:YAG laser therapy. We analyzed data from these patients, including their age, BMI, smoking habits, presence of hypertension, alcohol consumption, and duration of IC treatment, aiming to find any correlations with the results of the vulvodynia test or the NRS-11. However, no significant correlations were found in any of the categories.

Reviewing the existing literature, the causes of vulvodynia and IC/BPS remain unknown, and no studies have identified any significant correlation with conditions such as hypertension or diabetes.

Based on our study findings, we propose that in cases where IC/BPS and vulvodynia coexist without other comorbidities, there is a potential for worsening of these conditions due to the interaction between the urinary and genital organs, rather than systemic factors. Therefore, directing attention to vulvodynia in IC/BPS patients who have not responded to bladder-targeted treatments alone could potentially lead to the discovery of more appropriate and effective treatment strategies.

In our study, we evaluated the therapeutic effects of combined VEL + Nd:YAG laser treatment on the vagina and labia. Significant improvements were observed in the NRS-11 scores over time. At T0, the initial score was 9.53±0.62, which decreased to 4.33±0.47 at T06 and further decreased to 1.33±0.47 at T12 (p<0.01 in both cases). In a previous study that utilized VEL treatment alone, NRS-11 scores improved from 9.11±0.92 to 2.09±2.03 at 0 to 12 months, respectively (p<0.01) [6].

Comparing our results with the previous study, we found that IC/BPS patients who did not respond to bladder treatment alone achieved greater improvement in NRS-11 scores with our combined VEL + Nd:YAG approach. Although a direct comparison is not feasible due to differing conditions, the addition of Nd:YAG laser treatment appeared to enhance the improvement in NRS-11 scores.

Previous reports focusing on genitourinary syndrome of menopause (GSM) have also demonstrated similar findings, with sexual pain, a primary symptom of GSM, showing greater improvement with the VEL + Nd:YAG approach compared to VEL treatment alone [16]. Moreover, this improvement exhibited long-lasting effects [17].

Similarly, our study demonstrated positive outcomes in ICSI and ICPI scores. The ICSI score decreased from 15.73±1.77 at T0 to 1.73±0.77 at T12, while the ICPI score decreased from 14.33±1.07 at T0 to 1.26±0.92 at T12. In a previous study using VEL treatment alone, the ICSI score changed from 15.6±1.33 at T0 to 5.67±2.17 at T12, and the ICPI score changed from 13.0±1.50 at T0 to 4.78±0.67 at T12 [6]. These findings suggest that the addition of Nd:YAG laser further enhanced the improvements in ICSI and ICPI scores.

Overall, our results support the notion that simultaneous treatment of the vagina and external genitalia is more effective when both genital and urinary diseases coexist. VEL + Nd:YAG laser treatment demonstrated beneficial effects in improving NRS-11, ICSI, and ICPI scores, with the addition of Nd:YAG laser appearing to enhance the therapeutic effects in this context. It is worth noting that Nd:YAG laser treatment has been reported to be effective in addressing pain in various fields [18-20], not limited to genital conditions [17].

To further investigate the impact of treating the external genitalia for bladder symptoms, we analyzed the changes from T0 to T06 (Δ_T0-6_) and from T0 to T12 (Δ_T0-12_) in various parameters. Table 2 displays the correlation between the changes in the vulvodynia test and the corresponding parameters. Notably, only the Δ_T0-6_ values of NRS-11 (p=0.007) and PUF (p=0.020) showed a significant correlation with the Δ_T0-6_ in the vulvodynia test while the other parameters did not demonstrate a significant correlation.

This indicates that in the short term, there is a strong correlation between the improvement in NRS-11 scores and the improvement in Vulvodynia test scores. Treating both genital and bladder pain together seems to have a synergistic therapeutic effect.

However, in the long term, there was no significant correlation observed between the vulvodynia test and the assessed parameters. ChatGPT-4 explained that this phenomenon suggests that the long-term impact of treating IC/BPS and vulvodynia may involve independent improvements in each symptom, influenced by multiple factors.

Given that there is no previous research investigating the treatment progress of patients with both IC/BPS and vulvodynia, the findings from our study provide new insights into this specific patient population.

We can provide potential evidence to support why we observed therapeutic effects in both vulvodynia and IC/BPS in our study. The shared developmental origin of genitourinary tissues may contribute to the coexistence of IC/BPS and vulvodynia [21]. Mast cells have been observed in both conditions, and their activity may stimulate pain-transmitting neurons [22,23]. The innervation of the female urinary and genital systems is governed by the same sacral nerve pathways [24,25]. Endocrine dysregulation may play a role in IC/BPS and vulvodynia [26,27]. Targeting vulvodynia in IC/BPS patients could lead to more effective treatment strategies. Further research is needed to understand the interaction between these systems.

Our study utilized chatGPT-4 for efficient and accurate data analysis. By following the methods suggested by chatGPT-4, we discovered a difference in the healing progress of the bladder and genitals in patients experiencing pain in both areas. Given that IC/BPS and vulvodynia involve multiple factors contributing to pain, we believe that utilizing chatGPT-4 to choose analysis methods can be effective.

## Conclusions

Our study, despite its limitations due to the extremely small sample size and the known symptomatic variability of the syndrome, provides evidence supporting the significant therapeutic effects of the combined VEL + Nd:YAG laser treatment for patients with vulvodynia and IC/BPS. The inclusion of laser treatment targeting the vagina and external genitalia resulted in enhanced outcomes compared to VEL treatment alone. Short-term correlations between improvements in pain scores and the vulvodynia test demonstrated an initial synergistic effect. Importantly, our results showed improvements at both six and 12 months, suggesting sustained benefits. However, it is worth noting that long-term improvements in each symptom may occur independently, potentially influenced by various factors. These findings contribute valuable insights into the treatment of patients with coexisting vulvodynia and IC/BPS, highlighting the significance of comprehensive approaches. Further research, including comparative studies between VEL + Nd:YAG laser and VEL laser alone, is warranted to evaluate treatment efficacy and gain a better understanding of the interaction between these conditions, ultimately leading to the development of more effective strategies.
